# Dreams: The Mind’s Minecraft

**DOI:** 10.7759/cureus.61561

**Published:** 2024-06-03

**Authors:** Abdulqadir J Nashwan, Abdelaziz Hendy, Ahmad A Abujaber

**Affiliations:** 1 Nursing and Midwifery Research Department, Hamad Medical Corporation, Doha, QAT; 2 Nursing Department, Ain Shams University, Cairo, EGY; 3 Nursing Department, Hamad Medical Corporation, Doha, QAT

**Keywords:** simulation, sleep, dreaming, minecraft, dreams

## Abstract

Minecraft is a game known for its limitless potential for creation, allowing players to construct elaborate structures, explore vast landscapes, and encounter a variety of creatures and scenarios, all within a controlled, virtual environment. Similarly, our dreams are constructed by the subconscious mind, using the “blocks” of memories, emotions, and sensory experiences accumulated during waking life. This editorial highlights the intricate relationship between the dream worlds created in sleep and the virtual landscapes we explore in Minecraft, highlighting how both territories are constructed from the building blocks of our subconscious mind. It emphasizes the role of dreams as simulators for real-life events, particularly in mitigating potential risks, much like Minecraft allows players to engage in risk-free exploration and problem-solving within its pixelated universe. In addition, this editorial aims to illuminate the functions of dreams in memory consolidation, emotional processing, and brain development while showcasing the importance of creativity and imagination in enhancing our mental health and understanding of reality.

## Editorial

The interpretation of dreams

The human fascination with dreams stretches back throughout history; however, formal dream theory is a relatively recent development. Early civilizations often attributed dreams to divine messages or glimpses into the future. It was not until the late 19th century that Sigmund Freud, the father of psychoanalysis, revolutionized dream theory with his book entitled “The Interpretation of Dreams” (1899) [1]. Freud believed dreams were a “royal road to the unconscious,” where hidden desires and anxieties manifested themselves symbolically. This theory sparked a new interest in dream analysis, though Freud’s emphasis on repressed desires has been challenged by later theorists.

The 20th century saw the emergence of new dream theories that diverged from Freud’s psychoanalytic approach [2-6]. In 1953, Hall’s cognitive theory proposed dreams as a way for the brain to process recent experiences and consolidate memories [2]. Meanwhile, the discovery of rapid eye movement (REM) sleep in the 1970s led to the activation-synthesis theory, which suggests that dreams are the brain’s attempt to make sense of random neural activity during that sleep stage [2,3]. Dream theory continues to evolve, with various perspectives offering insights into the complex world of dreams [4-6].

While the exact purpose of dreams remains debated, several functions have been proposed. Some theories suggest that dreams aid in memory consolidation, weaving new experiences into existing memories [7]. Others posit that dreams serve as a form of emotional processing, helping us regulate emotions or deal with difficult experiences [7]. There is also evidence that dreams might play a role in brain development and physical health by restoring neurochemicals and preparing the brain for potential threats [8].

In the “universe” of sleep, our brains craft worlds as vivid and complex as those built in the pixelated landscapes of Minecraft. This popular video game, released in 2011, allows players to build and explore virtual landscapes using textured cubes and serves as a useful metaphor for understanding the whimsical and intricate world of our dreams [9]. This editorial explores the fascinating parallels between our dreams and the sandbox game of Minecraft, where creativity and simulation blend into an experience that both entertains and enlightens.

Building blocks of the mind

Minecraft is known for its limitless potential for creation, which allows players to construct elaborate structures, explore vast landscapes, and encounter a variety of creatures and scenarios, all within a controlled, virtual environment [10]. Similarly, our dreams are constructed by the subconscious mind, using the “blocks” of memories, emotions, and sensory experiences accumulated during waking life. Just as Minecraft players manipulate blocks to create new realities, our minds rearrange these elements into dream sequences that can be bizarre, whimsical, or even surreal.

Dreams as simulations

Similar to Minecraft, where scenarios can be manipulated and outcomes tested without real-world consequences, dreams often simulate real-life events. The scientific community, led by researchers like Antti Revonsuo, posits that one function of dreaming might be to simulate threatening events, allowing us to rehearse responses to potential dangers [11,12]. Threat simulation theory simply mentions that dreaming serves an evolutionary function by simulating threatening events [11]. Dreams activate threat detection and response mechanisms, enhancing survival skills [11]. It suggests that repetitive dream threats improve problem-solving and adaptive responses in waking life [12]. Therefore, this idea parallels Minecraft gameplay, where players can engage in combat with enemies or navigate treacherous environments safely within the game’s interface of survival mode.

Navigating the uncharted

Dreams often take us to places we have never been, creating roads and maps to worlds that do not exist outside our minds [13]. Minecraft echoes this experience by generating endless, ever-changing terrains that players can explore. The randomness and variability of dream landscapes reflect Minecraft’s algorithmically generated environments, where no two maps are exactly alike. Exploring these landscapes in dreams and Minecraft can lead to unexpected discoveries and challenges, prompting adaptation and problem-solving. Interestingly, Dr. Hirt’s research explores how indigenous dreaming practices, integrated as sources of geographical information, enhance participatory mapping with Indigenous communities, potentially serving as a valuable source of geographical and cartographic data [14].

The element of surrealism

Both dreams and Minecraft have inherent surrealism (an artistic and literary movement that seeks to express the imaginative world of dreams and the unconscious mind by transcending everyday, logical reality. It often combines unlikely images and scenarios in bizarre and fantastical ways [15]). In dreams, physics and logic are often defied; one might imagine flying over a cityscape or talking to a long-lost relative in an impossible setting [16]. Minecraft also embraces this whimsy, with its blocky aesthetics and simple yet expansive laws that govern its universe, allowing for experiences that defy the mundane constraints of the real world.

To sum up, dreams, much like a session of Minecraft, are our mind’s way of constructing, exploring, and navigating complex worlds using the building blocks of our consciousness. They allow us to simulate scenarios, explore uncharted psychological territories, and even indulge in the surreal. Understanding this parallel can provide insights into the importance of play and imagination for our mental health and daily lives. Whether dreaming or gaming, we perpetually build, explore, and reshape our understanding of reality.
